# Geographical information system (GIS) modeling territory receptivity to strengthen entomological surveillance: *Anopheles* (*Nyssorhynchus*) case study in Rio de Janeiro State, Brazil

**DOI:** 10.1186/s13071-018-2844-2

**Published:** 2018-04-19

**Authors:** Hermano Gomes Albuquerque, Paulo Cesar Peiter, Luciano M. Toledo, Jeronimo A. F. Alencar, Paulo C. Sabroza, Cristina G. Dias, Jefferson P. C. Santos, Martha C. Suárez-Mutis

**Affiliations:** 1 Laboratório de Doenças Parasitárias, Fiocruz, Av. Brasil, 4365 Manguinhos, Rio de Janeiro, RJ 21040-360 Brazil; 2 Laboratório de Monitoramento Epidemiológico de Grandes Empreendimentos, Fiocruz, Av. Brasil, 4365 Manguinhos, Rio de Janeiro, RJ 21040-360 Brazil; 3 Laboratório de Diptera, Fiocruz, Av. Brasil, 4365 Manguinhos, Rio de Janeiro, RJ 21040-360 Brazil; 4 Secretaria de Estado de Saúde do Rio de Janeiro, R. México, 128 Centro, Rio de Janeiro, RJ 20031-142 Brazil

**Keywords:** Malaria, GIS, Geoprocessing, Vector, *Anopheles*, Receptivity, Surveillance

## Abstract

**Background:**

Extra-Amazonian malaria mortality is 60 times higher than the Amazon malaria mortality. Imported cases correspond to approximately 90% of extra-Amazonian cases. Imported malaria could be a major problem if it occurs in areas with receptivity, because it can favor the occurrence of outbreaks or reintroductions of malaria in those areas. This study aimed to model territorial receptivity for malaria to serve as an entomological surveillance tool in the State of Rio de Janeiro, Brazil. Geomorphology, rainfall, temperature, and vegetation layers were used in the AHP process for the receptivity stratification of Rio de Janeiro State territory.

**Results:**

The model predicted five receptivity classes: very low, low, medium, high and very high. The ‘very high’ class is the most important in the receptivity model, corresponding to areas with optimal environmental and climatological conditions to provide suitable larval habitats for *Anopheles* (*Nyssorhynchus*) vectors. This receptivity class covered 497.14 km^2^ or 1.18% of the state’s area. The ‘high’ class covered the largest area, 17,557.98 km^2^, or 41.62% of the area of Rio de Janeiro State.

**Conclusions:**

We used freely available databases for modeling the distribution of receptive areas for malaria transmission in the State of Rio de Janeiro. This was a new and low-cost approach to support entomological surveillance efforts. Health workers in ‘very high’ and ‘high’ receptivity areas should be prepared to diagnose all febrile individuals and determine the cause of the fever, including malaria. Each malaria case must be treated and epidemiological studies must be conducted to prevent the reintroduction of the disease.

## Background

Malaria is an infectious disease of epidemiological relevance in Brazil, where the Amazon region is the main endemic area, accounting for 99.8% of all cases [1]. However, the malaria mortality rate outside the Amazon region was 60 times higher than in the Amazon in 2013 [2]. Cases recorded outside the Amazon, usually in the Atlantic Forest biome, may be indigenous (or autochtonous) or imported, with the latter representing most of the cases, 739 (or 89.4%) in 2013 [2]. Conceptual model of malarious areas consider these imported cases as a factor of vulnerability [3]. Imported malaria cases can be diagnosed at any location, depending on the patient’s destination, and could become a problem in municipalities outside of endemic areas, as healthcare professionals in those regions may not have experience with the diagnosis and treatment of malaria [2]. In particular, imported malaria could be a major problem if the cases occur in areas where local vectors and environmental conditions favoring malaria transmission [3] are present [4] and in our conceptual model it’s defined as receptivity. In Brazil, the presence of vector species in genus *Anopheles*, subgenus *Nyssorhynchus*, outside of the Amazon can favor the occurrence of outbreaks or reintroductions of malaria in those areas [1, 2, 5].

The World Health Organization (WHO) advocates the importance of monitoring direct and indirect factors that determine malaria transmission in malaria-free areas [5], categorizing malaria foci into six classes: endemic; residual active; residual non-active; cleared-up; new potential; and new active (or pseudo). The State of Rio de Janeiro is categorized as a new potential focus, due to its receptivity to malaria, the history of cases and the possibility of reintroduction from imported cases. The identification and mapping of all potential *Anopheles* larval habitats, especially those of *Nyssorhynchus* mosquitoes [6, 7], is essential for successful malaria vector control and to change the status of Rio de Janeiro from new potential to cleared-up. In such cases, it is universally recommended the use of geographical information systems (GIS) as a tool for epidemiological and entomological surveillance [3, 5].

According to the “Guidelines on the elimination of residual foci of malaria transmission” [5], malaria foci are determined by the presence of parasite, host and vector populations. Entomological surveillance is an important tool to determine the receptivity to malaria in malarious areas [5], defined as areas “in which transmission of malaria is occurring or has occurred during the preceding three years” [8]. In addition, investigation of the vulnerability to infection is an important part of surveillance efforts in malarious areas to determine the magnitude of the malariogenic potential.

Thus, this study aimed to construct a territory receptivity model of malaria focusing on potential larval habitats and on the aquatic hemi-population of *Anopheles* subgenus *Nyssorhynchus* to serve as an entomological surveillance tool in the State of Rio de Janeiro. Malaria vector populations consist of two different phases, aquatic and airborne, referred to as ‘hemi-populations’ [9]. This study examined the aquatic hemi-population of *Anopheles* (*Nyssorhynchus*).

## Methods

### Study area

The State of Rio de Janeiro comprises 92 municipalities, with a population estimated at 15,989,929 inhabitants in 2010, distributed over 43,780 km^2^. The state borders three other states: Minas Gerais, São Paulo and Espírito Santo [10]. Their influence area includes 264 municipalities. The state capital, Rio de Janeiro, includes one of the country’s major ports and the second busiest international airport, allowing for large movements of people, which are important to the dynamics of imported malaria.

**Fig. 1:**
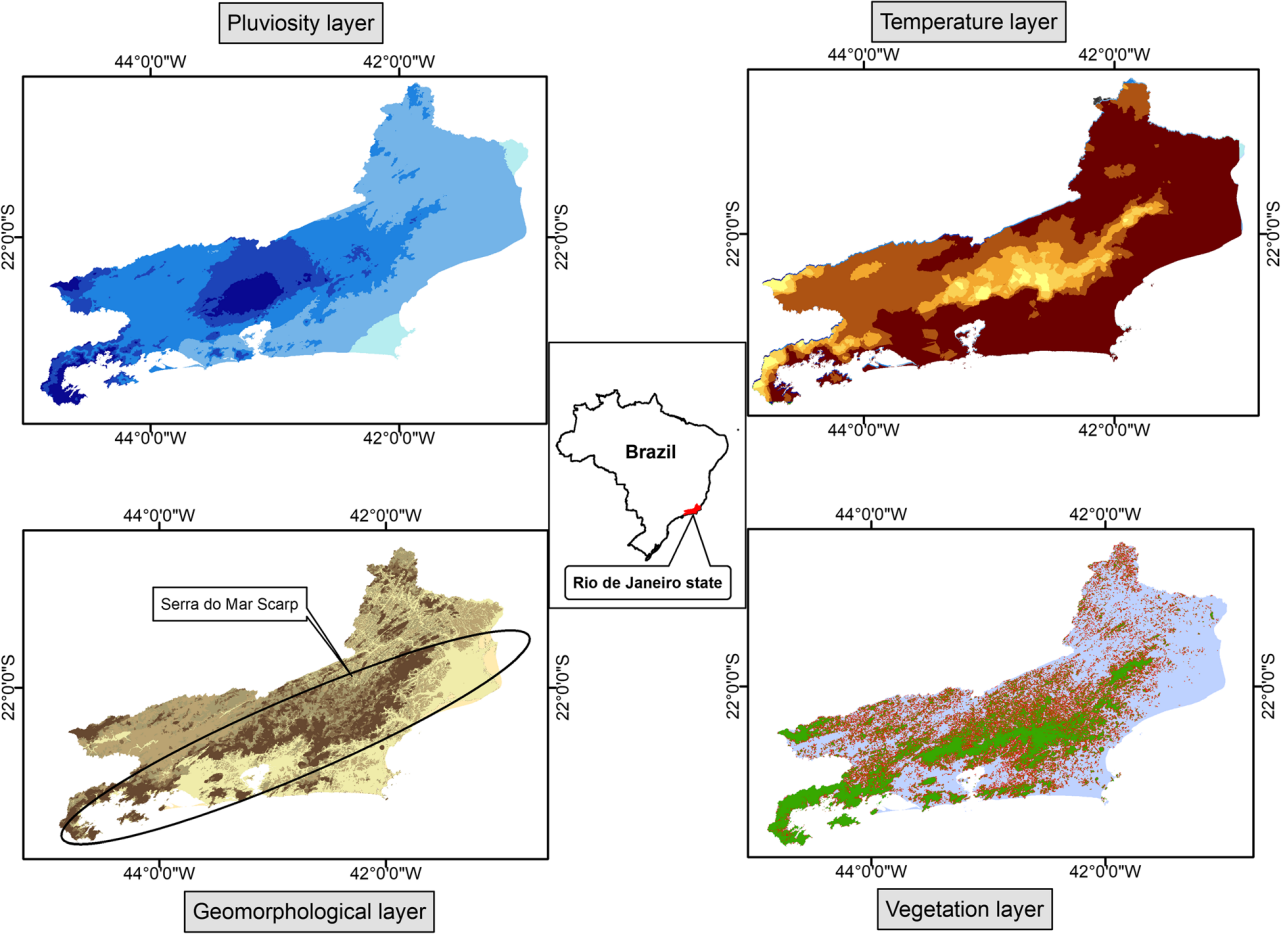
Four layers of the AHP model: pluviosity, temperature, geomorphology and vegetation

The geomorphology of Rio de Janeiro is very diverse, with hills, scarped mountains, isolated mountains and lowlands (Fig. 1). The territory is divided by the Serra do Mar mountain range, forming two main hydrological regions, North and South/Southeast [11]. The vegetation consists mainly of Atlantic forest, which covers approximately 30% of the state’s area. Deforestation has occurred at a slower rate in Rio de Janeiro than in other Atlantic forest regions because of the high slopes of the Serra do Mar range. These two geographical features (vegetation and geomorphology) influence the hydrological behavior in the State of Rio de Janeiro. High precipitation levels are observed along the Serra do Mar range [11]. Northwest and central coastal regions contain the largest water bodies of the state, whereas the lowlands adjacent to the Serra do Mar have many smaller water bodies. Thus, Rio de Janeiro has many different geomorphological landscapes that provide suitable larval habitats for *Anopheles* vectors.

### Database and data analysis

The receptivity model was constructed using secondary data. The study database used information from “The status of environment” study by the Rio de Janeiro State Institute for the Environment (Instituto Estadual do Ambiente - INEA, 2010) and the Rio de Janeiro State Department of the Environment (Secretaria de Estado do Ambiente - SEA). A database was created to describe the current environmental context of Rio de Janeiro State, providing support to solve political, environmental and social problems. Four information layers of the database were used in the study: geomorphology, rainfall, temperature and vegetation (Fig. 1). The features of the model were based on the study by Dlamini et al. [12] and are collectively referred to as potential larval habitat [13, 14]. This study pinpoints the importance of identification and mapping the waterbodies, which are the places for ovoposition of many species of *Anopheles*, as a strategy for successful vector control, especially larval source management. All the variables that compose the authors model have strict relation to the ideal conditions to the existence of *Anopheles* larval habitats.

Data analysis was conducted with the Analytic Hierarchy Process (AHP) [15]. This method entails classifying and assigning weights to each layer of the study model. First, each layer was categorized into classes based on their frequency distribution. The geomorphology layer was categorized into six classes relative to sea level: 0–20 m; 20–100 m; 100–200 m; 200–400 m; > 400 m; and sandy areas. Rainfall (ranging between 812–2834 mm) was divided into five classes: 812–1000 mm; 1000–1300 mm; 1300–1500 mm; 1500–1700 mm; and 1700–2834 mm. The average monthly temperatures ranged between 9.19–23.15 °C, and were also divided into five classes: 9.19–15 °C; 15–17 °C; 17–19 °C; 19–21 °C; and 21–23.15 °C. A 200 m buffer was incorporated around the vegetation layer as a potential area for presence of *Anopheles* (*Nyssorhynchus*) (Fig. 1).

Two entomologists with extensive experience in malaria vectors were interviewed for a pairwise comparison of model layers. The weights assigned to each layer class are shown in Table 1. The geomorphology layer was weighted more heavily in the model because it represents the most structural component of the larval habitat in the region, affecting all other components (45%). The geomorphology of a region does not change over ecological time, and thus areas with optimal conditions for larval development in the past remain the same in the present. The climate components, temperature and rainfall distribution, were weighted equally (20%) and a little higher than the vegetation component (15%), because they are macroscale determinants that vary by latitude, longitude, altitude and proximity to the ocean [16]. The vegetation component affects the environment on a microclimatic scale through its influence on the hydrological cycle and the presence of water bodies [17, 18].

**Table 1:** Layer weight AHP model

Information layer	Influence on the model (%)	Classes/categories	Weight on model
Temperature (°C)	20	9.15–15.0	1
		15.1–17.0	2
		17.1–19.0	3
		19.1–21.0	4
		21.1–23.15	5
Pluviosity (mm)	20	812–1000	1
		1001–1300	2
		1301–1500	3
		1501–1700	4
		1700–2834	5
Geomorphology (m)	45	Sandy áreas	5
		0–20	5
		21–100	4
		101–200	3
		201–400	2
		> 401	1
Vegetation	15	200 m outside vegetation (Buffer)	5
		Vegetation cover	2
		Other áreas	1

Model processing was done in ArcGIS Desktop 10.0 (Environmental Systems Research Institute, Redlands, CA, USA), using Spatial Analyst Tool and Weighted Overlays to construct the AHP model. This tool allows overlaying several layers/databases using a common measurement scale and weights each layer according to its importance.

After the AHP model was constructed, the distribution of receptivity classes was investigated in each municipality of the State of Rio de Janeiro. The layer of municipalities was obtained from the Brazilian Institute of Geography and Statistics (IBGE) website. The data were processed in ArcGis 10 using the Intersect tool to compute a geometric intersection between input data layers.

The Rio de Janeiro State has a total area of 43,780 km^2^, but the total area projected for the state in the model was 42,182 km^2^. The proportions of receptivity class cover were calculated using the model’s total area. The discrepancy in total area is due to the database used in the study.

## Results

**Fig. 2:**
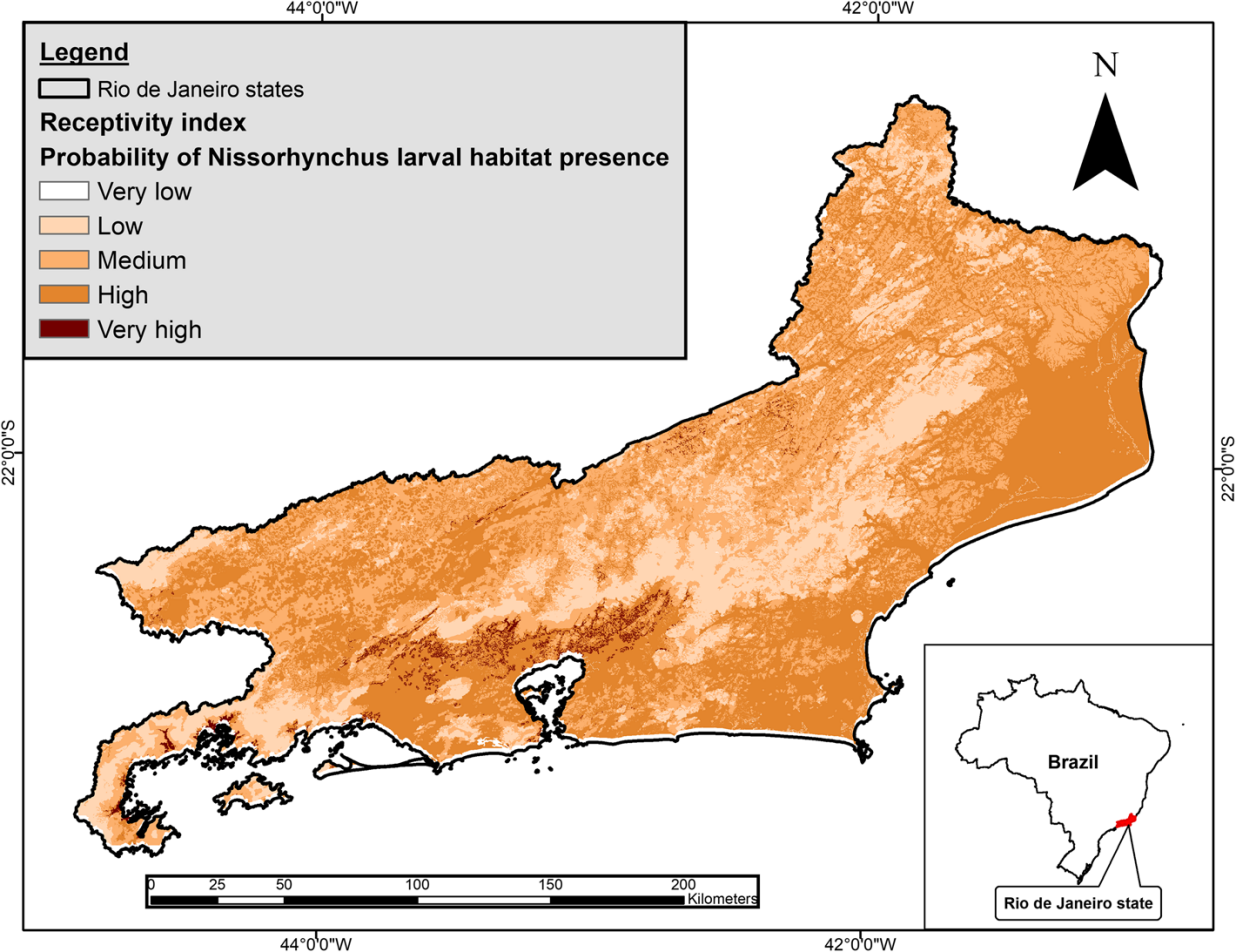
Receptivity map - Probability of *Anopheles* (*Nyssorhynchus*) larval habitat presence in Rio de Janeiro State

The model predicted five receptivity classes: very low; low; medium; high; and very high. The ‘high’ class covered the largest area, 17,557.98 km^2^, or 41.62% of the area of Rio de Janeiro State, whereas the lowest cover (0.35 km^2^ or 0.08% of the state’s area) corresponded to the ‘very low’ class (Table 2, Fig. 2). The ‘very high’ class is the most important in the receptivity model, corresponding to areas with optimal environmental and climatological conditions to provide suitable larval habitats for *Anopheles* (*Nyssorhynchus*) vectors. This receptivity class covered 497.14 km^2^ or 1.18% of the state’s area.

**Table 2:** Results of the *Anopheles* (*Nyssorhynchus*) larval habitat model in Rio de Janeiro State, by classes

Receptivity class	Cover area (km^2^)	Cover area (%)
Very low	1.08	0.002
Low	9302.8	20.6
Medium	15,770	36
High	18,020	41.2
Very high	601.7	1.4
No data	0.42	0.798
Total	43,696	100

Rio de Janeiro has 92 municipalities, of which 41 have ‘very high’ receptivity areas, but only 11 municipalities have more than 10 km^2^ of ‘very high’ receptivity areas. Of those municipalities, Duque de Caxias (66.71 km^2^, 14.25% of its territory), Cachoeiras de Macacu (61.07 km^2^, 6.39%), Guapimirim (54.31 km^2^, 15.04%) and Magé (49.64 km^2^, 12.77%) have the highest absolute area of ‘very high’ receptivity areas, whereas the municipalities with the highest proportion of ‘very high’ receptivity areas are Japeri (25.91%), Guapimirim (15.04%), Duque de Caxias (14.25%), and Magé (12.77%). All of these municipalities are situated in the central region of Rio de Janeiro, with an estimated population of 1,280,903 [10].

## Discussion

The malaria receptivity index successfully stratified the State of Rio de Janeiro into different areas. In our study, receptivity refers to the potential presence of larval habitats for *Anopheles* (*Nyssorhynchus*) vectors. Thus, the higher the probability of an area having potential larval habitats of *Anopheles* (*Nyssorhynchus*) vectors, the higher the receptivity index, which ranged from 1 to 5 or from ‘very low’ to ‘very high’. The term larval habitat, which is defined as a “site at which developmental stages of mosquitoes (eggs, larvae, pupae) are found, including sites that appear to be ecologically suitable for particular species”, has recently been replaced by the terms breeding sites or breeding places [14]. This new term refers to the concept of species tolerance and the need for specific abiotic conditions, which represent the idea of ecological niche [13].

The ‘high’ receptivity class was the most common in the model and covered more than two-fifths of the state’s area (41.62%). This result can be explained by the extensive areas of lowlands in the state, which accumulate water from mountain rivers due to the low slope of the terrain. These areas contain water bodies that are potential larval habitats for *Anopheles* (*Nyssorhynchus*) mosquitoes [19, 20].

The areas classified as ‘very high’ are characterized by the highest temperatures and precipitation levels of the state and are mostly located in the lowland regions (0–20 m). The model for *Anopheles* (*Nyssorhynchus*) larval habitats also included a forest component. Forest fragments have the potential to retain rainfall water [18]. This ground water is gradually released into the rivers favoring the existence of permanent water bodies in the region. Moreover, tropical forests have the potential to affect the climate conditions in adjacent areas [17]. The stable climatic conditions near forest fragments favor larval development and the occurrence of suitable larval habitats.

Most ‘very high’ receptivity areas are concentrated in the central and south regions of the state. The ‘very high’ areas in the central region are situated mainly in lowlands surrounding the south hillside of the Serra do Mar range, in an area known as Baixada Fluminense. Malaria incidence rates in Baixada Fluminense were high in the past [19, 21, 22]. In fact, this region was one of the three most malaria-endemic areas of the state at the beginning of the 20th century [23], and our results showed that it still provides suitable larval habitats for *Anopheles* (*Nyssorhynchus*) vectors, remaining receptive to malaria. The south region also has many ‘high’ receptivity areas, in the region known as Costa Verde.

Some municipalities had a significant probability of having larval habitats for *Anopheles* vectors in a large proportion of their territory. Moreover, three of the municipalities with ‘very high’ receptivity areas have had autochthonous cases confirmed between 2002–2010 (Cachoeiras de Macacu: eight cases; Rio de Janeiro: seven cases; and Paraty: three cases) [22], which provides further support to our study model. In these areas, WHO [3] recommends that entomological surveillance should be conducted on a permanent basis to understand the environmental dynamics and prevent malaria outbreaks from imported or introduced cases, or malaria reintroduction.

Additionally, imported malaria cases were recorded in some municipalities with an elevated proportion of ‘very high’ receptivity areas: Nova Iguaçu (24 cases), Duque de Caxias (24 cases), and Cachoeiras de Macacu (six cases) [22]. According to our model, these municipalities are among the five most receptive municipalities in the State of Rio de Janeiro. This fact is of high epidemiological relevance, because the combination of receptivity and vulnerability (i.e. importation risk) can create the perfect conditions for the emergence of outbreaks or disease reintroduction. In a review of the literature, Cohen et al. [24] detected 75 malaria resurgence events in 61 countries, showing the real possibility for that kind of event to occur in places where malaria was endemic in the past and that are still receptive to the disease. The main factor linked to malaria resurgence events (68/75 events, 91%) was the weakening of malaria control programs, for which the main reason was disruptions in funding.

It is important to emphasize that our model focused on the geographical features of *Anopheles* distribution described in the literature. Soberón & Peterson [25] proposed a model to determine the distribution of a species with four classes of factors corresponding to biotic factors, abiotic conditions, the regions accessible to dispersal, and evolutionary adaptability. In the current study, only abiotic factors were considered, and the model did not include *Anopheles* samples. Nevertheless, every model has limitations and is a simplification of reality, which does not invalidate it as a way of reflection about the object it analyzes. Furthermore, our model fit with the areas that were endemic in Rio de Janeiro State in the past. This kind of approach can be useful in areas without recent occurrence records of *Anopheles* species and in regions with a history of endemicity but without continuous investment in malaria control.

## Conclusions

We used freely available databases for modeling the distribution of receptivity areas for malaria transmission in the State of Rio de Janeiro. This was a new and low-cost approach to support entomological surveillance efforts. Health workers in ‘very high’ and ‘high’ receptivity areas should be prepared to diagnose all febrile individuals and determine the cause of the fever, including malaria. Each malaria case must be treated, and epidemiological studies must be conducted to prevent the reintroduction of the disease. A study about vulnerability to imported malaria cases is necessary to complete the malarious areas analysis in Rio de Janeiro State.

Finally, GIS and geoprocessing technologies are important to many areas, including health and territorial planning. Using available databases, many models can be constructed for different purposes, transforming raw data into relevant information. Nevertheless, modeling is a way to simplify the complexity of reality and not a goal in itself [26].

## Abbreviations

AHP: Analysis hierarchical process
GIS: Geographic information system
IBGE: Instituto Brasileiro de Geografia e Estatística (Brazilian Instituto of Geography and Statistics)
INEA: Instituto Estadual do Ambiente (Environment State Institute)
SEA: Secretaria de Estado do Ambiente (Environment State Secretary)
WHO: World Health Organization
